# Correction: Tetramerizing tGCN4 domain facilitates production of Influenza A H1N1 M2e higher order soluble oligomers that show enhanced immunogenicity *in vivo*

**DOI:** 10.1016/j.jbc.2021.100407

**Published:** 2021-03-02

**Authors:** Sweety Samal, Tripti Shrivastava, Praveen Sonkusre, Zaigham Abbas Rizvi, Rajesh Kumar, Shubbir Ahmed, Preeti Vishwakarma, Naveen Yadav, Manish Bansal, Kanchana Chauhan, Sebanta Pokhrel, Supratik Das, Padmakar Tambare, Amit Awasthi

VOLUME 295 (2020) PAGES 14352–14366

In the present article, it was stated in the Results section that: “All the proteins transiently expressed either in HEK293T or Expi293F™ cells as described in experimental procedures except for M1-3xΔtGCN4 construct. No expression was seen for M1-3xΔtGCN4 (the experiment was repeated three times).” Both instances of “M1-3xΔtGCN4” should read “M2-3xΔtGCN4.”

Additionally in the article it was stated in the Experimental procedures section that: “The synthetic gene was constructed by linking two peptide sequences originating from the human influenza consensus sequence SLLTEVETPTRSEWESRSSDSSD (Peptide-1) and SLLTEVETPIRNEWGSRSNGSSD (Peptide-2).” It should read: “The synthetic gene was constructed by linking two or three peptide sequences originating from the human influenza consensus sequence SLLTEVETPTRSEWESRSSDSSD (Peptide-1), SLLTEVETPIRNEWGSRSNGSSD (Peptide-2) and SLLTEVETPTRSEWESRSSGSSD-(Peptide-3) linked with GG linker, C terminally tGCN4 as described previously.” The sentence “the third construct consists of one repeat of peptide 1 and two repeats of the peptide 2 and other domains similar to M1-2x.” should read “the third construct M2-3x consists of one repeat of peptide 1, one repeat of the peptide 2, one repeat of peptide 3 and other domains similar to M1-2x as shown in supporting information.”

Also, it was stated in the Experimental procedures section that: “Immunization was carried out three times (1, 14, and 28 days).” It should read: “Immunization was carried out three times (0, 21, and 42 days).”

